# Single-cell DNA sequencing identifies risk-associated clonal complexity and evolutionary trajectories in childhood medulloblastoma development

**DOI:** 10.1007/s00401-022-02464-x

**Published:** 2022-07-13

**Authors:** Marina Danilenko, Masood Zaka, Claire Keeling, Stephen Crosier, Stephanie Lyman, Martina Finetti, Daniel Williamson, Rafiqul Hussain, Jonathan Coxhead, Peixun Zhou, Rebecca M. Hill, Debbie Hicks, Vikki Rand, Abhijit Joshi, Edward C. Schwalbe, Simon Bailey, Steven C. Clifford

**Affiliations:** 1 Wolfson Childhood Cancer Research Centre, Translational and Clinical Research Institute, Newcastle University Centre for Cancer, Newcastle upon Tyne, UK; 2 National Horizons Centre, Teesside University, Darlington, UK; 3 School of Health and Life Sciences, Teesside University, Middlesbrough, UK; 4 Department of Neuropathology, Royal Victoria Infirmary, Newcastle University Teaching Hospitals NHS Foundation Trust, Newcastle upon Tyne, UK; 5 Department of Applied Sciences, Northumbria University, Newcastle upon Tyne, UK; 6 Biosciences Institute, Newcastle University, Newcastle upon Tyne, UK

**Keywords:** Medulloblastoma, Paediatric cancer, Heterogeneity, Clonal evolution

## Abstract

We reconstructed the natural history and temporal evolution of the most common childhood brain malignancy, medulloblastoma, by single-cell whole-genome sequencing (sc-WGS) of tumours representing its major molecular sub-classes and clinical risk groups. Favourable-risk disease sub-types assessed (MB_WNT_ and infant desmoplastic/nodular MB_SHH_) typically comprised a single clone with no evidence of further evolution. In contrast, highest risk sub-classes (*MYC*-amplified MB_Group3_ and *TP53*-mutated MB_SHH_) were most clonally diverse and displayed gradual evolutionary trajectories. Clinically adopted biomarkers (e.g. chromosome 6/17 aberrations; *CTNNB1*/*TP53* mutations) were typically early-clonal/initiating events, exploitable as targets for early-disease detection; in analyses of spatially distinct tumour regions, a single biopsy was sufficient to assess their status. Importantly, sc-WGS revealed novel events which arise later and/or sub-clonally and more commonly display spatial diversity; their clinical significance and role in disease evolution post-diagnosis now require establishment. These findings reveal diverse modes of tumour initiation and evolution in the major medulloblastoma sub-classes, with pathogenic relevance and clinical potential.

**Supplementary Information:**

The online version contains supplementary material available at 10.1007/s00401-022-02464-x.

## Introduction

Medulloblastoma (MB) is the most common malignant brain tumour of childhood. Approximately 30% of patients die from their disease and survivors are left with life-long disease and treatment-associated morbidities [31]. Diversity between individual MBs has long been recognised, encompassing established disease molecular groups (i.e. MB_WNT_, MB_SHH_, MB_Group3_ and MB_Group4_) and subgroups (i.e. MB_Group3-4_ subgroups I–VIII) with distinct clinico-molecular characteristics, alongside well-defined genomic features at the bulk-tumour level [3, 14, 22, 30, 31, 42, 43]. Critically, this inter-tumoural diversity has been exploited clinically; specific molecular sub-classes define risk-associated therapy stratification in current clinical trials. MB_WNT_ tumours, and infant MB_SHH_ tumours with desmoplastic/nodular (DN) pathology, are associated with high survival rates (i.e. favourable-risk) and receive reduced-intensity therapies, while *MYC*-amplified MB_Group3_ and *TP53*-mutated MB_SHH_ carry the highest risk and gravest prognoses [14, 31].

The potential presence of clinically significant cellular heterogeneity within individual MBs was first suggested by studies which demonstrated intra-tumoural variation of specific genetic defects at the single-cell level (e.g. *MYC* amplification [8, 11, 41]). Moreover, multiple sampling of bulk MB tumours has suggested spatial heterogeneity of genetic markers within individual tumours, questioning whether a single diagnostic biopsy captures all necessary genetic information for use in clinical management [28]. Systematic single-cell studies of MB are essential to further explore these issues. Studies to date have, however, focussed on intra-tumoural transcriptional variability and its relationship to cellular origins and cerebellar development [15, 32, 46, 49], with only one specific study analysing genetic heterogeneity within two Li–Fraumeni-associated MBs [35]. The cellular genetic constituency of the major MB sub-classes, and its relationship to disease initiation, clonal and temporal evolution, thus remains poorly understood [8].

Here, we report the application of sc-WGS to reconstruct the development and clonal evolution of 14 MBs, encompassing sampling of two spatially distinct regions of each tumour. Unlike previous single-cell MB transcriptomic studies predicated on fresh tumour material [15, 32, 35, 46, 49], our use of fresh-frozen tumours enabled specific selection of molecularly and clinically phenotyped tumours representative of the major MB sub-classes and risk groups. Using these data, we derive a clinically focussed understanding of MB intra-tumoural heterogeneity, providing critical insights into differences in initiation, drivers, clonal evolution trajectories and spatial impact. Moreover, our findings highlight how understanding MB at the single-cell level offers significant potential to improve the clinical management of MB, through informing biomarkers for early disease detection, strategies for diagnostic sampling, and therapeutic targeting.

## Methods

### Sample selection

**Fig. 1:**
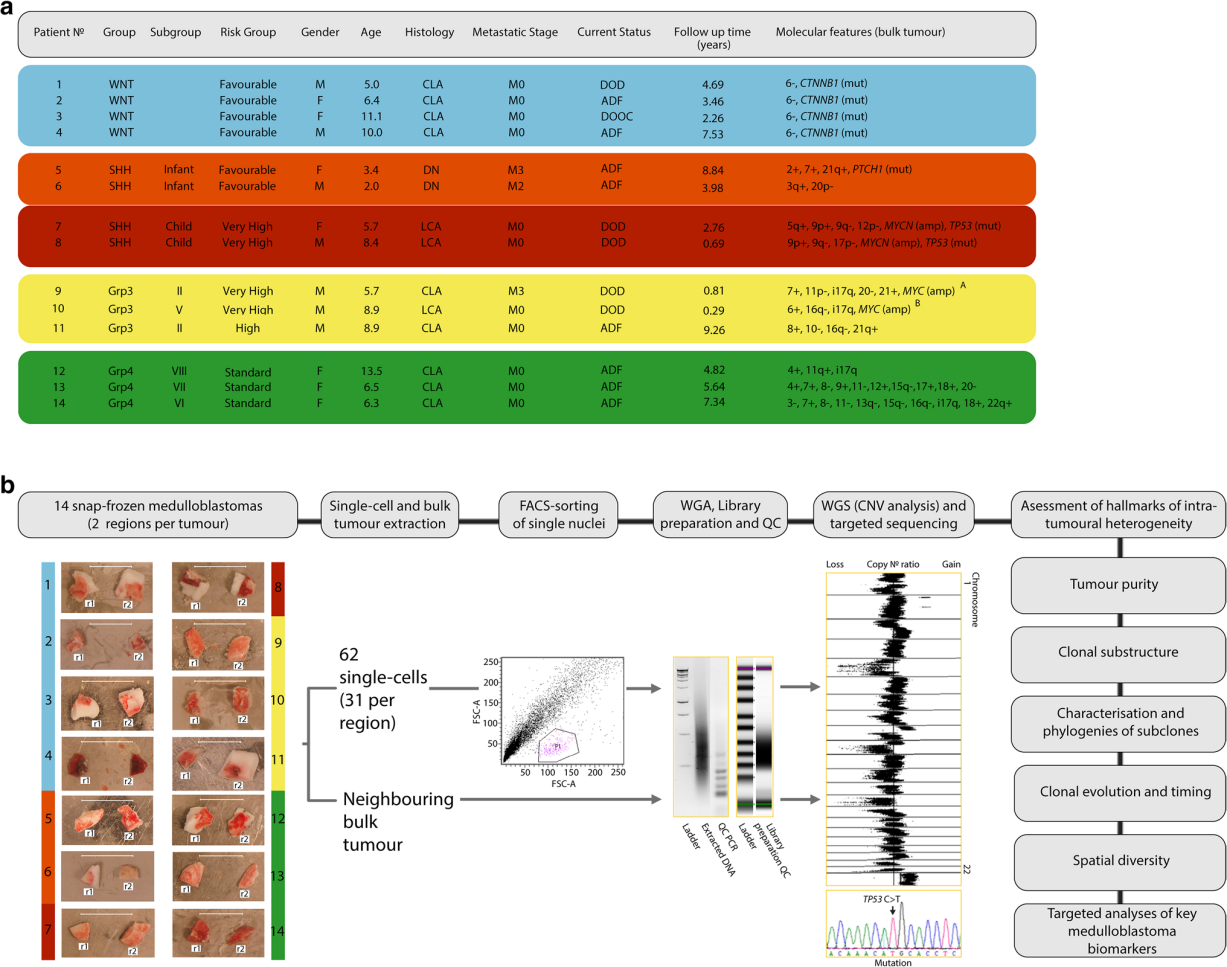
Single-cell analysis of childhood medulloblastoma: patient cohort and study design. **a** Selected cohort of 14 patients representing key medulloblastoma clinico-molecular sub-classes. Cohort encompasses all 4 molecular groups WNT (blue), SHH (light red—infant SHH and dark red—childhood SHH), Group3 (yellow) and Group4 (green), multiple subtypes and risk groups*. MYC*-amplified tumours with low A and high B percentages of amplified cells were selected for comparative analysis. **b** Study pipeline showing the main milestones. Scale bar on the tumour photographs equals 10 mm. Abbreviations: *LCA* large cell and anaplastic, *CLA* classic, *DN* desmoplastic/nodular, *amp* amplification, *mut* mutation, *FACS* fluorescence-activated single-cell sorting, *WGA* whole-genome amplification, *WGS* whole-genome sequencing, *CNV* copy number variant, *Grp* group, *r1/2*  region 1/2, *FCS* forward scatter, *DOD* dead of disease, *ADF* alive disease-free, *DOOC* dead of other cause

Fresh-frozen tumour material from 14 clinically annotated human primary MBs was selected for analysis, using extant molecular pathology data (Fig. 1; *TP53/CTNNB1* mutation, *MYC/N* amplification and DNA methylation array (molecular subgroup and copy number profiles)) [42]. Samples were collected with written, informed consent from UK Children’s Cancer and Leukaemia Group (CCLG, https://www.cclg.org.uk/) institutions and collaborating centres (study approval reference BS-2008–12). Tumour investigations were performed with approval from Newcastle/North Tyneside Research Ethics Committee (study reference 07/Q0905/71). Central review of histological variants was performed according to 2016 World Health Organization (WHO) criteria [22]. Metastatic status was determined according to Chang's criteria [4]. Patients < 4.0 years of age at diagnosis were regarded as infants. Tumours were assigned to the four consensus disease molecular groups (MB_WNT,_ MB_SHH,_ MB_Grp3_ and MB_Grp4_) [22] using established DNA methylation array-based methods [42]. Second-generation MB_SHH_ subgroups were assigned according to Schwalbe et al. 2017 [42]. Second-generation MB_Grp3/4_ subgroups [43] were assigned using the ‘Grp3 and Grp4 Classifier’ (https://www.molecularneuropathology.org/mnp/classifier/7). A cerebellar sample was obtained post-mortem from the Newcastle Brain Tissue Resource (https://nbtr.ncl.ac.uk/).

Two separate tissue pieces were selected from each tumour (received as two distinct surgical pieces), ranging from 2.4 × 2.6 mm (smallest) to 5.4 × 4.9 mm (largest) (Fig. 1) in size, to represent tissue fragment sizes typically used in diagnostic genetic analysis. For each region, histological sections confirmed material quality and 30 µm cryosections were produced for single-cell analyses; the remainder was used for comparative bulk tumour assessment.

### Bulk tumour analysis

For each tumour, DNA was isolated (DNeasy Blood and Tissue Kit (Qiagen, Cat No. 69504) from the two bulk tumour regions, combined and subjected to deep (180x) whole-exome sequencing (WES). Sequencing libraries (Agilent SureSelect Human All Exon kit (Agilent Technologies; Santa Clara, CA, USA)), fragmentation (hydrodynamic shearing system (Covaris, Massachusetts, USA)), product purification (AMPure XP system (Beckman Coulter, Beverly, USA)) and quantification (Agilent Bioanalyzer 2100 system), followed by Illumina paired-end sequencing (Illumina; San Diego, CA, USA), were performed according to the manufacturer’s instructions. Raw reads were mapped to the GRCh37/hg19 reference genome.

Chromosome arm-level and focal copy number variations (CNVs) were assessed using Nexus Copy Number 10.0 (BioDiscovery, El Segundo, CA, USA) and validated by visual inspection. Copy number segments were classified as balanced, gained (log2 ratio > 0.2) or lost (log2 ratio < − 0.2) [30]. Whole arm chromosomal changes were considered gained/lost when ≥ 90% of the probe signals exceeded defined thresholds [14].

Mutational analysis interrogated 192 genes previously either reported as frequently mutated driver genes in medulloblastoma or recognised to be acquired in relapsed disease [25, 29, 30, 38]. Next-generation sequencing datasets were analysed using Genome Analysis Toolkit (GATK) version 3.8, according to Broad Institute’s best practices (Burrows-Wheeler alignment, Samtools, Picard, Haplotype Caller, MuTect, ANNOVAR) [5, 10, 20, 21, 47]. Variants were predicted pathogenic if their consequence included coding or splice donor/acceptor mutations, ExAC and 1000 genomes frequency < 0.01, included stop codon or frameshift variants, were predicted to be deleterious by SIFT/Polyphen and FATHMM or were known mutations in MB [44]. Where germline data were available only somatic variants were reported. Variants with a bidirectional read depth ≥ 5 were further curated by visual inspection in Integrative Genomics Viewer (IGV) [39].

### Single-cell isolation and DNA sequencing

Single nuclei were isolated using standard methods and FACS sorting [2], followed by DNA extraction and whole-genome amplification (WGA) using the GenomePlex WGA4 kit (Sigma-Aldrich) according to the manufacturer’s protocol. DNA purification was performed according to Macaulay et al. [23]. An additional single-cell DNA quality control of WGA DNA fidelity by multiplex PCR-based analysis of multiple differentially sized amplification products was included as an adaptation to the protocol (Supplementary Methods Fig. 1). Cells with at least 4 products in multiplex PCRs were taken forward for library preparation.

Library preparation was performed using the Nextera XT 96-Index Kit (Illumina, cat. no. FC-131-1002) [23]. Shallow WGS was performed on NextSeq (Illumina), using 150 bp pair-end reads at the Sequencing Core Facility at Newcastle University to obtain target coverage of 0.15X for each single-cell library. Sequencing depth was determined from preliminary down-sampling experiments, as the optimal depth which produced findings consistent with both copy number aberrations detected at higher read-depths, and findings from parallel bulk tumour analysis (Supplementary Figs. 1 and 2). Data were processed using the CASAVA 1.8.1 pipeline (Illumina), and sequence reads were converted to fastq files. Sequencing reads from each single cell were demultiplexed using an in-house Perl script (demultiplex.pl) into independent fastq files, where each file represented the sequencing reads from one cell (https://emea.support.illumina.com/content/dam/illuminasupport/documents/documentation/ /bcl2fastq/bcl2fastq_letterbooklet_15038058brpmi.pdf). DNAs from 32 normal post-mortem cerebellar cells were sequenced as diploid controls, with 8 normal cells being sequenced per single-cell batch run.

### Single-cell data analysis

Eight hundred sixty-eight tumour nuclei were sequenced. Raw reads in the fastq format [6] were processed and quality-checked using FastQC (Babraham Bioinformatics). 865 tumour nuclei passed the initial QC step. Illumina adapters were trimmed and reads with Phred-scale base qualities of 10 and below were filtered out using Cutadapt [24]. Raw reads were mapped to human reference genome version GRCh37/hg19 using BWA-MEM algorithm of Burrows–Wheeler Aligner (BWA) [20] version 0.7.17. The output SAM files were converted to compressed BAM files using Genome Analysis Toolkit [26]. Alignment summary reports were generated using SAMtools [21] flagstat function (detailed data quality scores used for the final selection of cells are shown in Supplementary table 1). Base quality recalibration was performed according to GATK guidelines.

Copy number aberrations were assessed from processed*. Bam files using the CNVKIT copy number analysis pipeline [45]; an on-target bin size of 50 kb was used for optimal resolution and segmentation. Coverages were estimated by calculating the mean read depth for each genomic bin. The MAD (median absolute deviation) quality metric was used to assess the noisiness of sequencing data. Genomes with two standard deviations below or above the mean bin count were excluded from further analyses, leaving 430 nuclei for further assessment of copy number calls.

A panel of normal cells was compiled as a copy number reference from 72 high-quality diploid (normal) genome sequences. Copy number regions with segmented log2 ratio estimates of − 1 and 1 (circular binary segmentation, *p* < 0.00001 [33]) were assigned as single copy gain and loss, respectively. Copy number region smoothing was performed by merging adjacent segments with the same absolute copy numbers or regions with non-significant difference in segment ratios. Copy number outputs were validated by visual assessment in IGV browser. CNVs were taken forward for analysis if detected in > 2 cells and with consistent genomic coordinates. Putative driver genes within focal and subchromosomal CNV regions identified were assessed using the Cancermine database [19].

Tumour ploidy was inferred using copy number relative ratios [2]. For tumour purity assessment, cells derived from aneuploid tumours (from bulk analysis), but with entirely balanced diploid single-cell copy number states, were considered normal/non-tumour.

### Mutation analysis

Fifteen deleterious mutations observed in bulk tumour analysis were selected for further investigation in the single-cell DNA from their corresponding tumours using targeted Sanger sequencing, for inclusion in analyses of clonal composition and genomic evolution.

### Clone identification

Numeric matrixes consisting of copy number segments were derived from the final copy number calls for each normal and tumour single-cell genome (https://bioconductor.org/packages/release/bioc/html/CNTools.html). Matrices were clustered using Euclidean distances for each genomic bin, hierarchical clustering and Ward linkage. Optimal clusters were calculated using multiple methods as Elbow, Silhouette and Gap statistics. Clustering heatmaps were visualised using *heatmap.2* function of the gplot R package [48]. Clusters were further investigated using principal component analysis (PCA) using *k* = 2 dimensions and visualised applying the multidimensional scaling (MDS) R utility [7].

### (Sub)clonality measures

CNVs and mutations were assigned as clonal when detected in each identified CNV sub-clone within a tumour, and subclonal when only detected in certain subclones. Mutational analyses assumed dropout of one allele at random following single-cell WGA [16].

### Phylogenetic tree reconstruction

Neighbour-joining (NJ) trees were estimated from the *phylo* object constructed by computing pairwise Euclidean distances of the copy number segment numeric matrixes using *nj* function of *ape* R package [34]. All trees were re-rooted to a normal diploid cell matrix using the interactive *root.phylo* function. The final trees were plotted as downward-directed using *plot.phylo* function, and the edges were coloured according to the clone colour affiliations.

### Modelling tumour genomic evolution

Tree formation (hierarchical clustering) and optimal clone numbers (multidimensional analysis) were imputed to infer evolutionary models using the SCICoNE package [18]. Individual tumour data were visualised using the “fishplot” R package [27] (https://www.rstudio.com/). Tumours were assigned to three modes of evolution: (i) no evolution, characterised by acquisition of established driver events and structural chromosomal alterations at initiation, leading to the development of a single, stable rapidly expanding clone, (ii) punctuated evolution, established features and structural chromosomal aberrations are acquired early and further copy number events accumulate in a punctuated clonal burst, consistent with clone formation and gradual stable expansion, (iii) gradual (branching) evolution, where established features and structural chromosomal aberrations are acquired at initiation, followed by the parallel formation of clones and their subsequent branching [9, 12].

### Statistical analysis

Categorical variables were compared using Fisher’s exact tests, with a significance threshold of *p* < 0.05. *p* values were adjusted for multiple testing using “fdr” method (https://www.rdocumentation.org/packages/fda.usc/versions/2.0.2/topics/FDR). Analyses were performed using R.

## Results

### scDNA sequencing of medulloblastoma: patient selection and study design

To assess intra-tumoural genetic heterogeneity in the major sub-classes of MB, 14 primary tumours were selected, based on their bulk tumour profiles (Fig. 1a, Supplementary Figs. 1 and 2), to represent all four disease molecular groups and their characteristic genetic features: MB_WNT_, MB_SHH_ (infant and childhood subgroups), Group3 (subgroups II and V) and Group 4 (subgroups VI, VII and VIII). Tumours further represented the major molecular risk-groups in current clinical use [22, 31], encompassing favourable (MB_WNT_ and infant DN MB_SHH_), standard, high- and very high-risk tumours (*MYC*-amplified MB_Group3_ and *TP53*-mutated MB_SHH_) [42, 43] (Fig. 1a), and recently described whole-chromosome aberration phenotypes (i.e. Chr 7 + . Chr8- and/or Chr 11-; Goschzik et al. [14]). The study design and workflow are summarised in Fig. 1b.

### Tumour purity and risk-associated diversity in clonal substructure

**Fig. 2:**
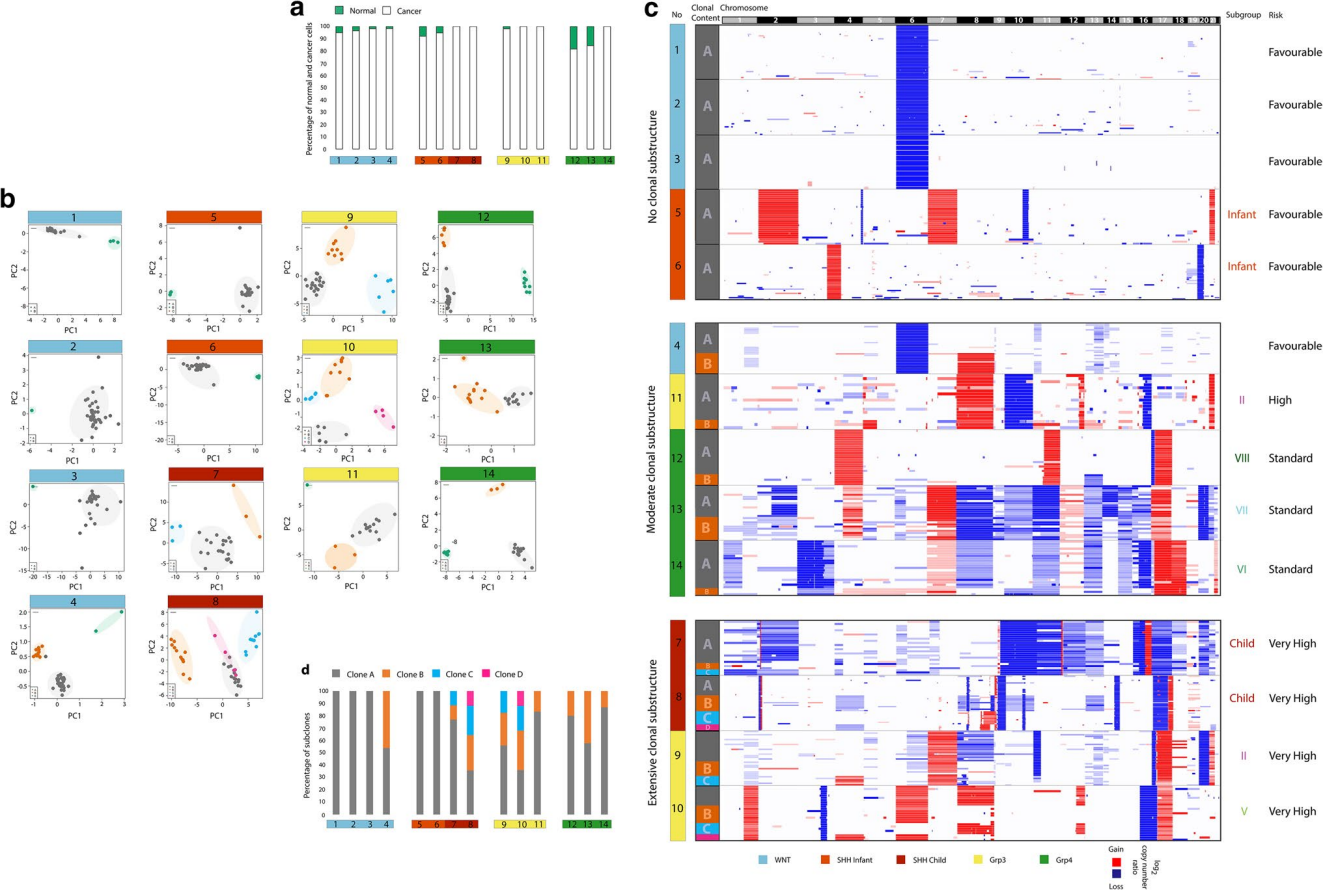
Tumour purity and clonal substructures in medulloblastoma. **a** Percentage of tumour (white; aneuploid) and normal (green; diploid) cells within each tumour. **b** Clonal sub-populations dissected by PCA/k-means clustering. Identified aneuploid clusters are labelled in grey, orange, blue and pink. Groups with a normal/diploid copy number profile are shown in green. **c** Clonal substructures dissected by hierarchical clustering (clustering dendrograms are presented in Supplementary Fig. 3). Three heatmaps represent the aneuploid copy number profiles for three patient groups with no, moderate or extensive clonal substructure. Single cells are plotted along the *y*-axis, and CNAs are plotted in genomic order along the *x*-axis. Clonal subpopulation identity (clone A, B, C or D), patient numbers and subgroup identifiers are shown on the left. Patient subtypes and risk profiles are indicated on the right. **d** Graph showing percentage of cells in each identified subpopulation. Abbreviations: *PC*  principal component

We sequenced 868 nuclei from the selected cohort (62 cells per tumour). After performing quality controls (see Methods section), 430 high quality single-nuclei datasets were taken forward for analysis. All tumours had a high tumour cell content (i.e. aneuploid; mean, 95%; range, 82–100% of cells), with low proportions of normal diploid cells evident (Fig. 2a). To dissect genetic clonal substructure within each tumour, PCA/k-means (Fig. 2b) and Euclidean distance (Fig. 2c; Supplementary Fig. 3) clustering were independently applied to the single-cell CNV data. The number and composition of dissected clusters were concordant across both approaches; in each case, normal diploid cells formed a separate cluster, while cancer cell CNV profiles revealed diverse structures between tumours, ranging from 1 to 4 distinct clusters or subclones (Fig. 2b–c).

Tumours could be assigned to 3 broad categories according to the number of sub-populations observed (Fig. 2b–d). First, low-complexity tumours with no evidence of genetic clonal substructure (*n* = 5), where all cells represented a single clone; notably, all were favourable-risk tumours (3 of 4 MB_WNT_ tumours examined and both infant MB_SHH_ tumours). Second, tumours with extensive clonal substructure (i.e. ≥ 3 sub-populations; *n* = 4); these were completely aligned with the very high-risk (VHR) tumours assessed (i.e. both *MYC*-amplified MB_Group3_ and both *TP53*-mutated/*MYCN*-amplified MB_SHH_). All tumours examined with high-risk LCA pathology (3/3) also fell within this group. Finally, a group of tumours with 2 sub-populations, which represented multiple sub-classes (WNT, Grp3 and Grp4) and different non-VHR risk profiles. Statistically, low-complexity, wholly clonal tumours were significantly associated with favourable-risk disease (5/6 vs. 0/8, *p* = 0.003, Fisher’s exact test), while the highest risk tumour sub-classes assessed were associated with the most extensive sub-clonality (4/4 vs. 0/10, *p* = 0.001, Fisher’s exact test). Importantly, cell numbers analysed were not associated with the number of subclones we identified (data not shown). Based on the mean number of 31 cells analysed per tumour, and the criteria we set for subclone detection (i.e. reproducible copy number patterns in 3 independent cells), we estimate that our approach was sensitive to detect subclones present at a frequency greater than approximately 10%.

### Uncovering clonal genetic composition and phylogenetic relationships

**Fig. 3:**
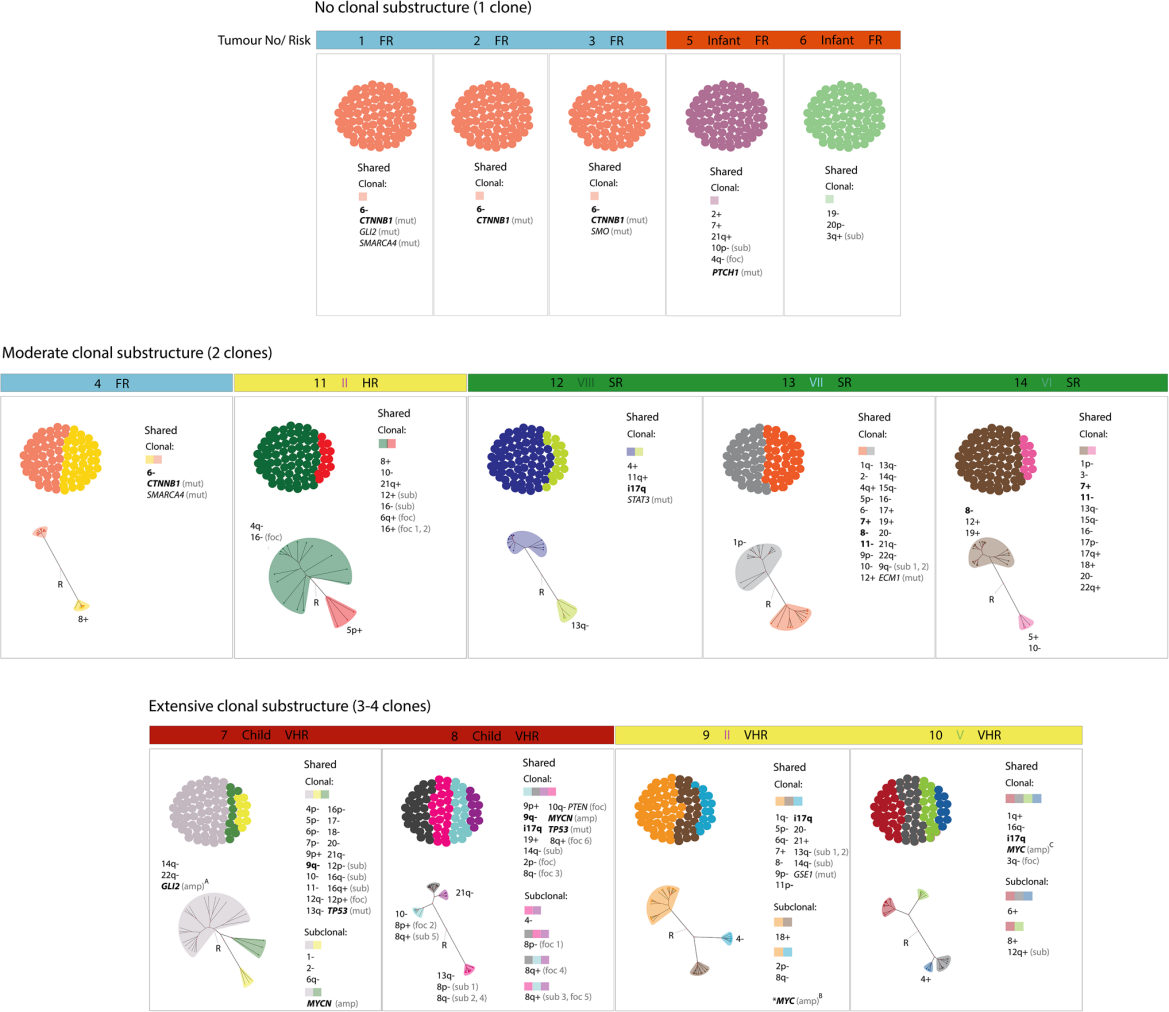
Content of clones and their phylogenetic inter-relationships. Clonal admixture plots for the three subclonal landscape categories are shown. Each distinct clone is represented in a different colour and proportional to the number of cells in which it was identified. Small, shaded squares and shaded labels on the phylogenetic trees represent the clone colour affiliation. CNVs and mutations defining more than one clone are listed beneath the shaded squares. Alterations unique to a single clone are labelled on the corresponding branches of phylogenetic trees. Established diagnostic and prognostic genomic features of medulloblastoma are shown in bold. *GLI2* amplification was detected in a single cell, but is illustrated as a key biomarker, A—*GLI2* amplification. B—*MYC* amplification was detected in 8/62 cells, but these did not pass QC and were excluded from analyses. C—*MYC* amplification in all clones. Abbreviations: *FR* favourable risk, *SR* standard risk, *HR* high risk, *VHR* very high risk, *foc* focal, *sub* subchromosomal

We dissected the CNV and mutational composition of each identified clone and their inter-relationships (Fig. 3, Supplementary Fig. 4, Supplementary Tables 2 and 3). As expected, the five tumours without evidence of substructure harboured a single homogeneous clone with shared genetic features. Marked clonal variation was observed within the remaining tumours; all displayed admixtures of clones with unique CNV and mutational content (Fig. 3). Applying neighbour-joining clustering, we determined the phylogenetic relationships between individual clones. In all cases, each identified subclone formed a distinct taxon but shared a common ancestor with other subclones in the same tumour (Fig. 3). Thus, identified clones were phylogenetically related, but the degree of relationship (i.e. the distances between different pairs of taxa) differed between tumours.

### Medulloblastoma evolutionary trajectories

**Fig. 4:**
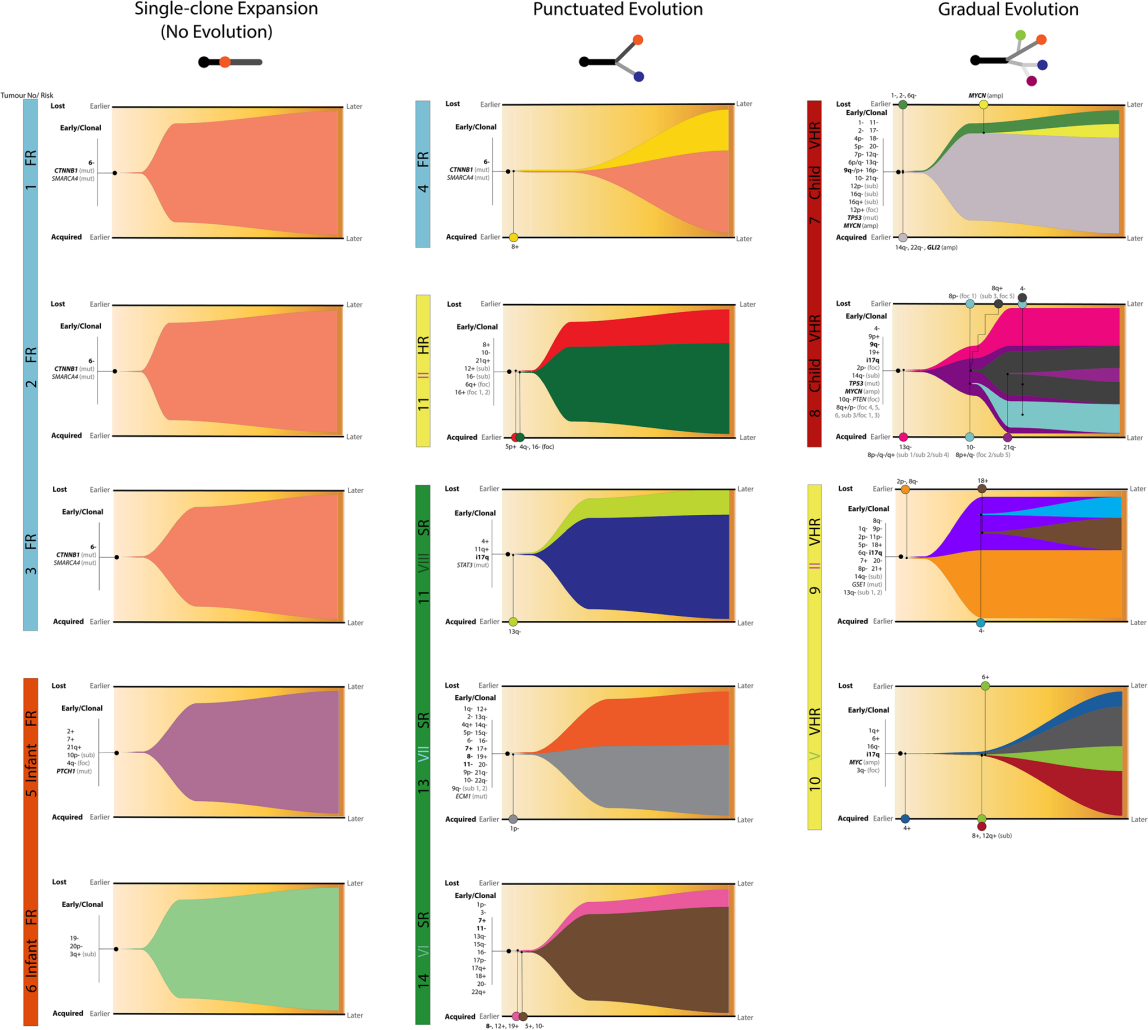
Evolutionary trajectories in medulloblastoma. Fish plots for groups of tumours associated with three different evolutionary trajectories. Plots display changes in clonal structure over pseudo-time, involving two time points, “Earlier” and “Later”. Consistent with previous figures, clonal admixtures are represented in the corresponding colours. Tumour initiating events, preceding subclone formation, are listed on the left (bracketed). Labelled shaded circles represent the events leading to subclone formation, and display their estimated time of origin. Acquired and lost alterations are shown at the bottom and top of the plot, respectively. The well-defined genomic biomarkers of medulloblastoma are shown in bold. See Figs. 1 and 3 for abbreviations

Modelling clonal evolution in our cohort using SCICoNE [18] revealed the three previously identified groups of tumours with different clonal substructures (Figs. 2c, 3) were associated with distinct evolutionary trajectories (*p* = 7.9 × 10^–7^; Fisher’s exact test) (Fig. 4). Favourable-risk tumours without clonal substructure showed no evidence of clonal genomic evolution (i.e. single-clone expansion), acquiring established driver events (e.g. *CTNNB1* mutation in MB_WNT_; *PTCH1* mutation in infant MB_SHH_) [31] and structural chromosomal alterations at initiation, associated with the development of a single, stable, rapidly expanding clone.

Tumours with a moderate clonal substructure followed a route of punctuated evolution. They obtained a number of established features (e.g. a combination of Chr7 + , Chr8– and Chr11− in the absence of gene-specific mutations) and structural chromosomal aberrations early in tumourigenesis [14, 31, 42]. The accumulation of further copy number events is then associated with a punctuated clonal burst, consistent with formation of both clones at similar time points, followed by gradual expansion and clonal stability over time.

The VHR tumours examined, with most extensive clonal substructures, best fitted a model of gradual (branching) evolution (Fig. 4). These tumours acquired high-risk drivers (e.g. *TP53* mutation, *MYC* and/or *MYCN* amplifications), alongside multiple structural CNVs at medulloblastoma initiation. Further accumulation and loss of CNVs is consistent with ongoing instability and formation of clones, followed by their subsequent branching. These findings are consistent with the diminution of some clones over time, while others gradually expand. This evolutionary trajectory shows the highest complexity and magnitude of events, consistent with their aggressive clinical behaviour and low survival rates [14, 31, 42].

### Clonality, detectability and timing of origin of individual events

**Fig. 5:**
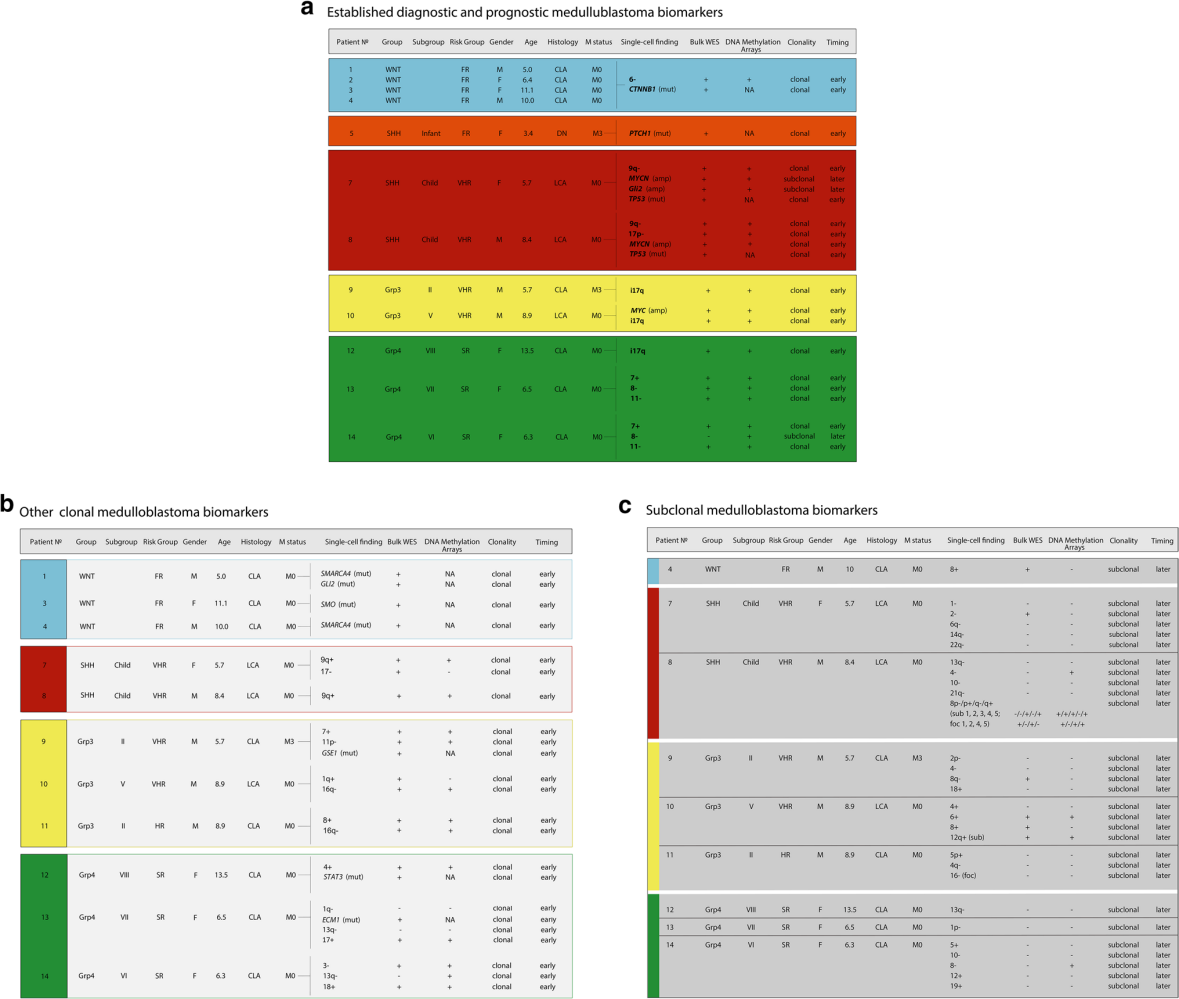
Clonality, detectability and timing of established medulloblastoma biomarkers (**a**) diagnostic and prognostic disease biomarkers in current clinical use, or supported by trials-based studies; shown in bold) [14, 31]. Summaries of other whole-arm/chromosome and mutational clonal (**b**) and subclonal (**c**) medulloblastoma biomarkers. Specific biomarkers were assessed only in their defined subgroup-specific context. See Fig. 1 for abbreviations

We next assessed individual CNV and SNV mutations (Fig. 3), their clonality, and its relationship to their detection at the sc-WGS and bulk tumour levels (WES and DNA-methylation array data) (Fig. 5). First, we investigated diagnostic and prognostic disease biomarkers in current clinical use [31], or supported by trials-based studies [14]; these were assessed strictly within their relevant subgroup-specific context (Fig. 3a). The overwhelming majority (20/23; 87%) of such biomarkers observed were clonal, emerged early at initiation and were detected in all three datasets examined. The remaining three instances were sub-clonal and occurred later (*MYCN* and *GLI2* amplifications in MB_SHH_ patient 7 and Chr8- in MB_Grp4_ patient 14). Other clonal whole-arm/chromosome CNVs detected are summarised on Fig. 5b. Importantly, a series of sub-clonal CNVs were detected at the single-cell level; the majority were observed in small proportions of cells (mean 31% of cells, range from 12 to 85% of cells) and occurred later in tumourigenesis. They were predominantly undetectable (22/37) in both bulk tumour datasets (WES and DNA methylation array; Fig. 5c). Subchromosomal (> 15 Mb) and focal (< 15 Mb) CNVs detected at the single-cell level, and associated with putative driver genes, are summarised in Supplementary table 4; 15/19 of these were detectable at the bulk tumour level. Notably, the resolution of our analysis allowed identification of smaller focal CNVs. The smallest focal gains/losses reproducibly detected at our sequencing resolution (i.e. 0.15x) were, respectively, a 2.5 Mb *GLI2* amplification (patient 7) and a 6.5 Mb deletion on chr8 (patient 8); the presence of both was confirmed in our bulk data sets. To conclude, 74% of the assessed CNVs (115/155) were clonal early events, thus, rather than occurring gradually over time, polyploidy is typically associated with medulloblastoma initiation.

### Spatial heterogeneity at the cellular level

**Fig. 6:**
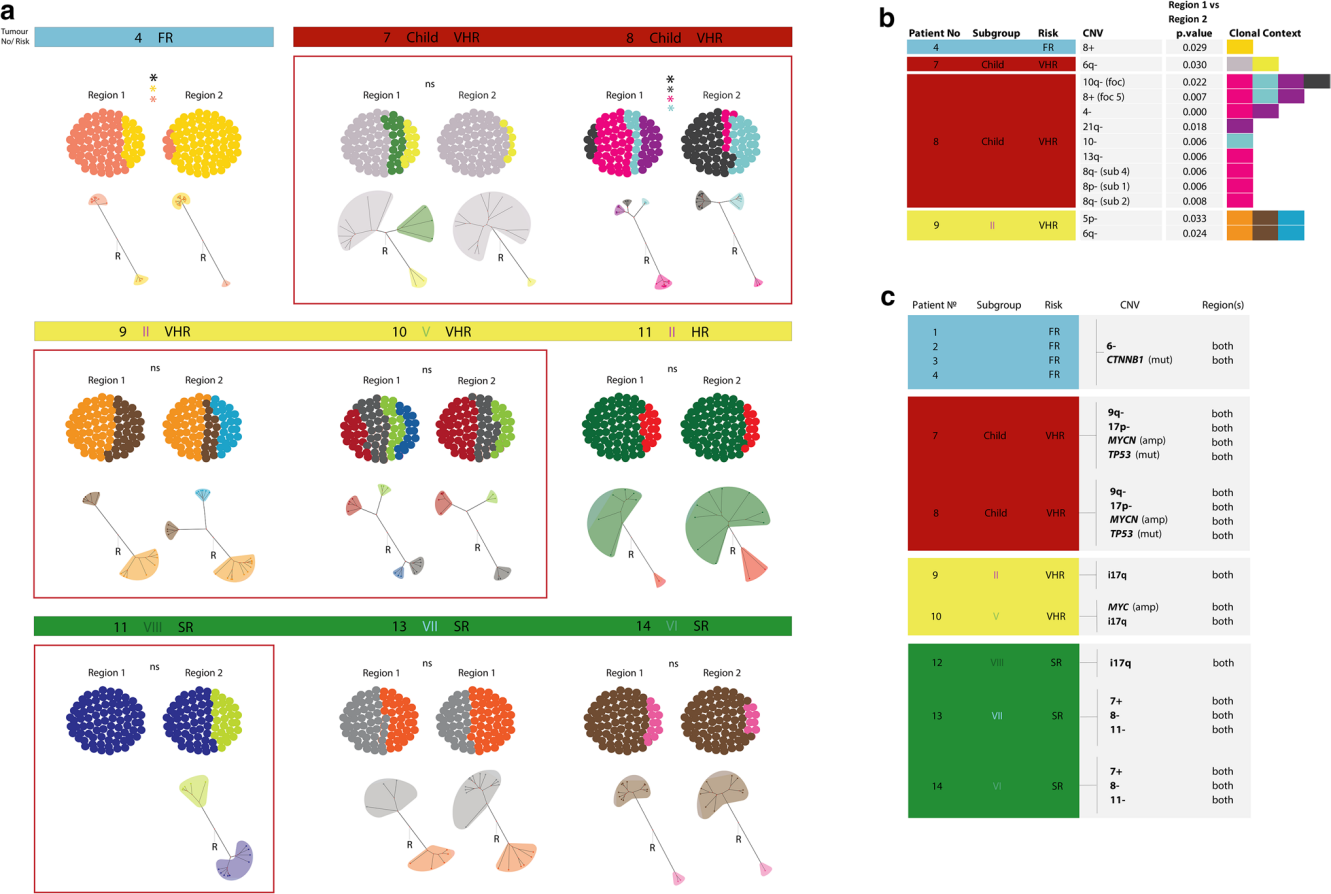
Spatial heterogeneity in medulloblastoma. **a** Clonal admixture plots and phylogenetic trees, reconstructed for two independent regions per tumour. Tumours with region-specific clones are framed in red. Significant differences in overall clonal composition between two regions are marked by black asterisks (*p* < 0.05 Fisher’s exact test), *ns* no significant difference; significant differences in the abundance of individual clones between two tumour regions is marked by clone colour-matching asterisks (*p* < 0.05, Fisher’s exact test). **b** Table summarises individual CNVs observed at significantly different frequencies between tumour regions (*p* < 0.05, Fisher’s exact test). Shaded colour blocks in each row represent the clones (coloured as on Figs. 3 and 6a) in which each CNV was detected. **c** Table summarises the regional distribution of established medulloblastoma biomarkers

To assess any spatial differences in clonal substructure, we created clonal admixture plots reflecting the percentage content of each clone within the two discrete areas of each tumour assessed (Fig. 6a). Of the 9 tumours with clonal substructure, 5 had a detectable clone which was only present in a single sampled region of the tumour, and clonal content was significantly different between regions in two tumours (Fig. 6a and Supplementary Table 5a, b). Region-specific clones formed distinct taxa within their tumour region on phylogenetic reconstruction (Fig. 6a). As expected, the five tumours with no evidence of clonal substructure did not display any spatial clonal variation (data not shown).

We further tested the distribution of individual genetic events across the two regions assessed within each tumour. The majority of CNVs (141/155) did not significantly differ in cellular frequency between the two tumour regions (Supplementary table 5C); 13 CNVs showed significant spatial variation (Fig. 6b). All of these were CNVs of unknown clinical significance. 14/15 tested SNV mutations were detected in both tumour regions (Supplementary Fig. 4). All assessed established medulloblastoma biomarkers were observed in both tumour regions, and at equivalent frequencies (Fig. 6c). *PTCH1* mutation and *GLI2* amplification were observed below our frequency threshold (i.e. ≥ 3 cells) in Patients 5 and 7, respectively, and thus were excluded from spatial analyses.

## Discussion

This study reveals diverse modes of initiation, clonal evolution and intra-tumoural genetic complexity between childhood MBs sampled from its major disease sub-classes and risk-groups, and provides a first understanding of their relationships to disease biology and clinical behaviour.

**Fig. 7:**
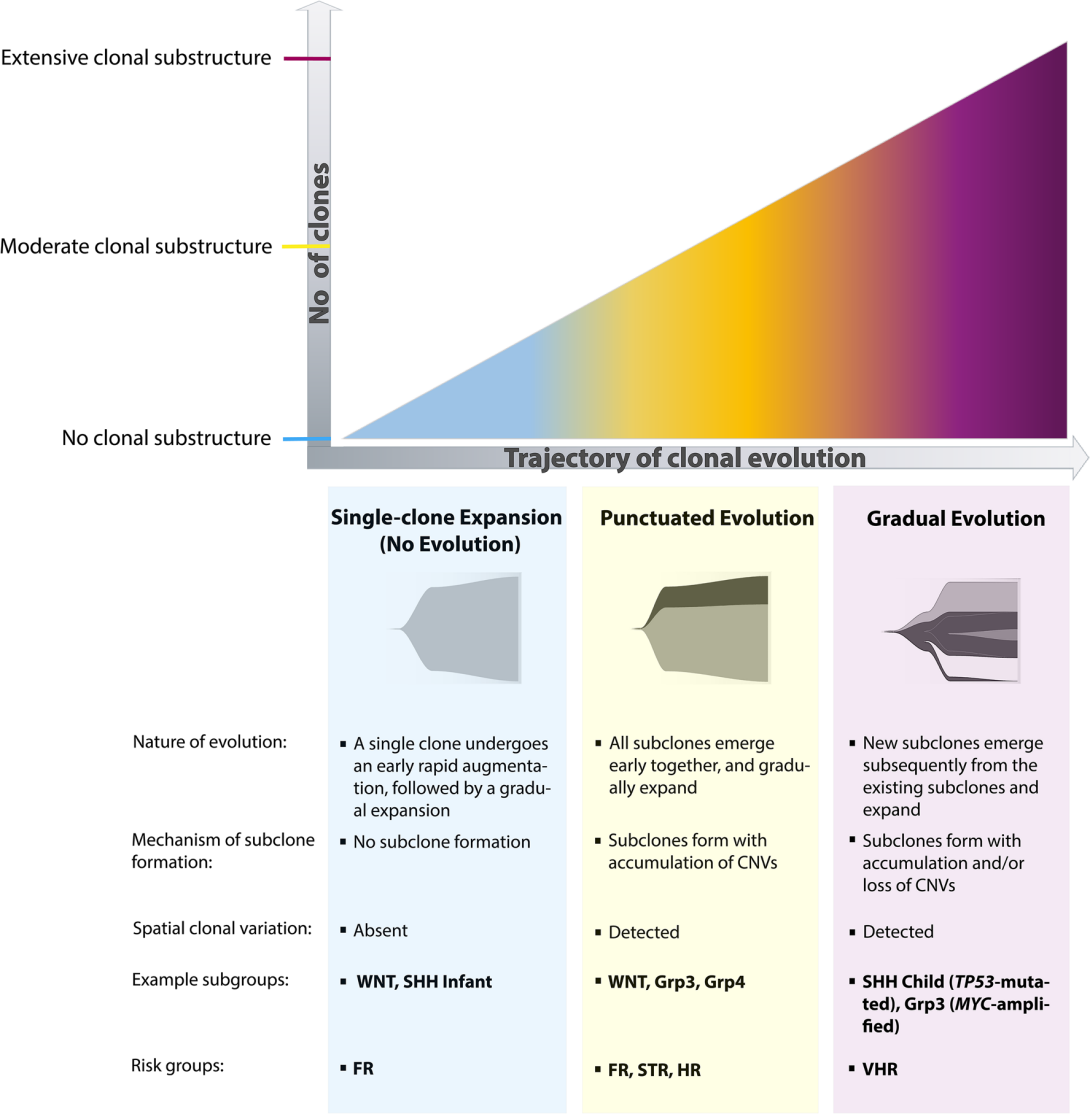
A model of risk-associated clonal evolution in medulloblastoma

Our discoveries have enabled definition of three distinct trajectories of disease initiation, development and clonal evolution. First, genetically homogeneous tumours with no clonal substructure, which acquire events at initiation (e.g. *CTNNB1* mutation and chromosome 6 loss in MB_WNT_) followed by expansion of a single clone (i.e. no evolution); such trajectories were only observed in favourable-risk disease groups examined (e.g. MB_WNT_, Infant MB_SHH_). Second, MBs with an extensive clonal landscape, characterised by multiple events at initiation, followed by the gradual evolution of subclones with unique genetic composition, resulting in the most diverse clonal substructures observed. These trajectories were only found in very high-risk tumours—their clonal diversity and clinical behaviour being consistent with the involvement of initiating events previously associated with genomic instability (*TP53* mutation and/or *MYC(N)* amplification [31, 35, 37])*;* and the presence of aggressive large-cell/anaplastic histology [11]. Independent inspection of single-cell WGS data reported for 2 Li–Fraumeni-associated primary MB_SHH_ tumours with *TP53* mutations [35] revealed evolutionary trajectories closely similar to the gradual trajectories we observed for our MB_SHH_-*TP53* mutated tumours, providing important validation of our findings for this tumour group. Thirdly, we also characterised an intermediate group of medulloblastomas with a moderate clonal composition, which were sampled from multiple non-VHR risk-groups. These typically acquired multiple events at initiation, followed by a punctuated evolutionary trajectory. From our findings, we proffer a model of genetic initiation, evolution, and clonal substructure for childhood medulloblastoma (Fig. 7). This model defines clearly distinct evolutionary paths along which disease evolution may proceed and which, while not absolute, provide a critical foundation for future investigations. Of note, we included a rare relapsing WNT patient (#1) in our investigation; this patient had clonal substructure equivalent to the other WNT tumours analysed, suggesting other factors play a role in WNT relapse.

Our findings have immediate clinical relevance. The majority of established medulloblastoma biomarkers examined (e.g. *CTNNB1* in MB_WNT_; *PTCH1/TP53/MYCN* in MB_SHH_; *MYC* in MB_Group3_; chr7 + /ch11- and i17q in MB_Group3/4_) [14, 31, 42] were revealed as early clonal/initiating events in tumour development, consistent with their driver roles in experimental models of spontaneous tumourigenesis, where tested [13, 17, 36, 40]. Their ubiquitous spatial presence highlights their potential importance for therapeutic targeting and informs requirements for tumour sampling in diagnostics and therapeutic stratification. Our data support the sufficiency of a single biopsy for the diagnostic assessment of established biomarkers, challenging previous recommendations that actionable targets found in a single biopsy are seldom clonal across the entire tumour [28]. Moreover, this finding highlights the potential of these key biomarkers of medulloblastoma initiation for use in cancer early detection and prevention strategies. For instance, clonal early events could be further investigated as potential early detection biomarkers in cerebrospinal fluid (CSF) or plasma circulating free DNA (cfDNA) screening. The early occurrence of multiple whole chromosome events suggests polyploidy commonly occurs as an early event in medulloblastoma development. Against this background, we additionally observed a series of previously unappreciated subclonal and/or region-specific events by sc-WGS, which were commonly undetectable in our WES analysis of the corresponding bulk tumour samples. Their biological and clinical significance now require investigation; to determine whether they play any role in further temporal evolution and resistance to therapy, or simply represent uninvolved ‘passenger’ events, will be of particular interest. In contrast to the aforementioned established biomarkers, multi-regional and/or single-cell sampling is essential for their detection.

Cellular transcriptional diversity has been previously observed within individual medulloblastomas [15, 46]; our data indicate this can arise against simpler genetic architectures. For instance, WNT tumours revealed here as genetically homogeneous, are associated with more diverse transcriptionally defined cellular sub-populations: four transcriptional meta-programs with variable underlying gene signatures were previously identified by single-cell transcriptome analyses of five WNT medulloblastomas [15]. Together, these findings suggest intra-tumoural differences in gene expression are influenced by non-genetic factors.

Our focus on tumours representing the major medulloblastoma disease sub-classes and molecular risk-groups is a significant strength of our study. However, we acknowledge, that future studies involving larger patient cohorts will be necessary to fully establish the relationship of clonal states to specific clinical and molecular disease features, and their translational relevance. The number of single cells investigated per tumour represents a potential limitation and it is possible that additional rare clones may be detected in deeper analyses; however, and importantly, cell number analysed was not associated with the number of subclones we identified in this study. Further studies will now be essential to explore these findings in larger clinically annotated cohorts, and with increased cell numbers. Furthermore, single-cell co-studies of genetic status and gene expression, ideally with longitudinal sampling over disease-course including at relapse, and in disease-relevant models, will be critical to discern their inter-relationships and relative contributions to tumour development, clinical behaviour and evolution.

In clinical therapeutics, our findings support a model whereby highest risk tumours may display greatest clonal diversity, which supports the hypothesis that clonal diversity increases potential for adaptation, evolution and escape under therapy, while favourable-risk tumour groups are least complex. Our findings are further supported by observations from a multi-regional bulk tumour sampling study, which demonstrated high-risk paediatric neuroblastomas, Wilms tumours, and rhabdomyosarcoma subtypes exhibit more frequent and extensive clonal branching [1]. Thus, in concert with morphological, molecular and cellular estimates currently used as prognostic markers, measures of clonality have potential as a common marker of prognosis in childhood cancers, which must now be considered and further investigated.

## Supplementary Information

Below is the link to the electronic supplementary material.

Supplementary Figures (DOCX 36778 KB)

Supplementary Table 1 Data quality metrics. Table provides information on DNA sequencing data quality (XLSX 187 KB)

Supplementary Tables 2 CNV calls from single-cell and bulk sequencing data. Tables summarize: (a) CNVs in the single-cell data; (b) Whole arm/chromosome CNVs identified in bulk WES data (XLSX 29 KB)

Supplementary Tables 3 A list of key mutations identified in the bulk WES and selected for validation in single-cell DNA. (**a**) Table summarises 16 mutations selected for validation (**b**–**e**) Summaries of Sanger sequencing results listed separately for (**b**) WNT, (**c**) SHH, (**d**) Grp3 and (**e**) Grp4 patients (XLSX 29 KB)

Supplementary Table 4 A summary of medulloblastoma driver genes covered by focal and sub-chromosomal CNV loci in the single-cell data. Table provides a list of focal and subchromosomal CNVs with identified driver gene loci. The functions of the genes are highlighted. The size, coordinates, and clonal status of CNVs are recorded (XLSX 21 KB)

Supplementary Tables 5 Statistical significance scores for the spatially resolved findings. Tables provide statistical significance scores: (**a**) for the proportions of cells contained in the clones of region 1 (R1) versus the proportions of cells in the clones of region 2 (R2) (**b**) comparing proportions of cells in certain individual clones in region 1 vs region 2 (c) for the individual CNVs, when comparing proportions of cells containing CNVs in region 1 versus region 2 (XLSX 36 KB)

## Data Availability

Data are available from the European Genome-Phenome Archive (EGA). Accession number is EGAS00001006392.
