# Supplementary material for: Single-cell DNA sequencing identifies risk-associated clonal complexity and evolutionary trajectories in childhood medulloblastoma development
Source: Acta Neuropathol. 2022 Jul 13;144(3):565–78. doi: 10.1007/s00401-022-02464-x (PMC9381458; doi:10.1007/s00401-022-02464-x)
Supplement: Supplementary file 1 — Supplementary Figures (DOCX 36778 KB) [file 401_2022_2464_MOESM1_ESM.docx]

**Supplementary Figures**

**Supplementary Methods Fig. 1**

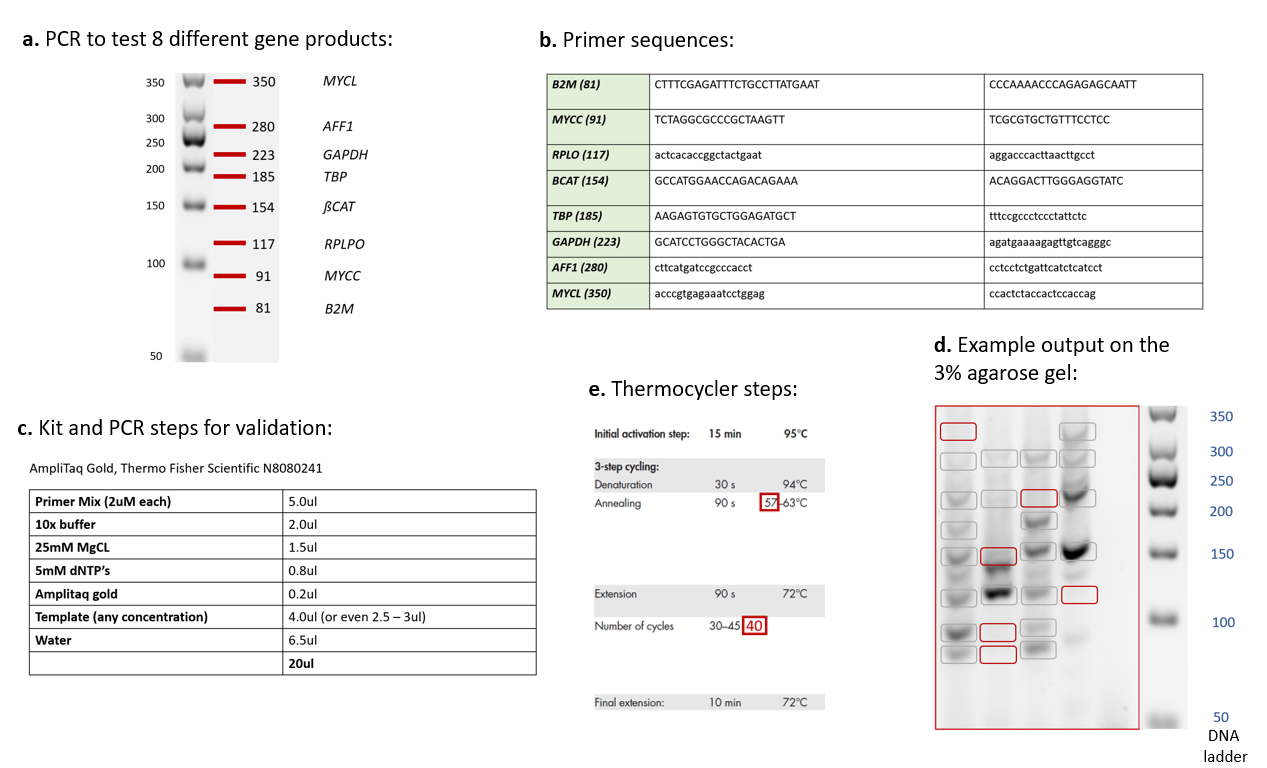


**Supplementary Methods Figure 1: Single-cell DNA quality control assessment protocol.** Figure shows the steps of the quality control protocol. **a)** 8 key gene products of different size were selected for PCR validation. **b)** Primers for these products were designed and validated in the laboratory. **c)** PCR kit applied and components of the reaction. **d)** PCR reaction steps. **e)** An output example on an agarose gel. Grey boxes highlight the presence of the expected product, red boxes indicate that the expected product is absent.

**

Supplementary Fig. 1.**

**Supplementary Fig. 1: Bulk tumour copy number dataset 1 for each patient.** Panels summarize the CNVs detected in each patient using DNA methylation arrays.



**Supplementary Fig. 2.**

**Supplementary Fig. 2: Bulk tumour copy number dataset 2 for each patient.** Panels summarize the CNVs detected in each patient using WES.


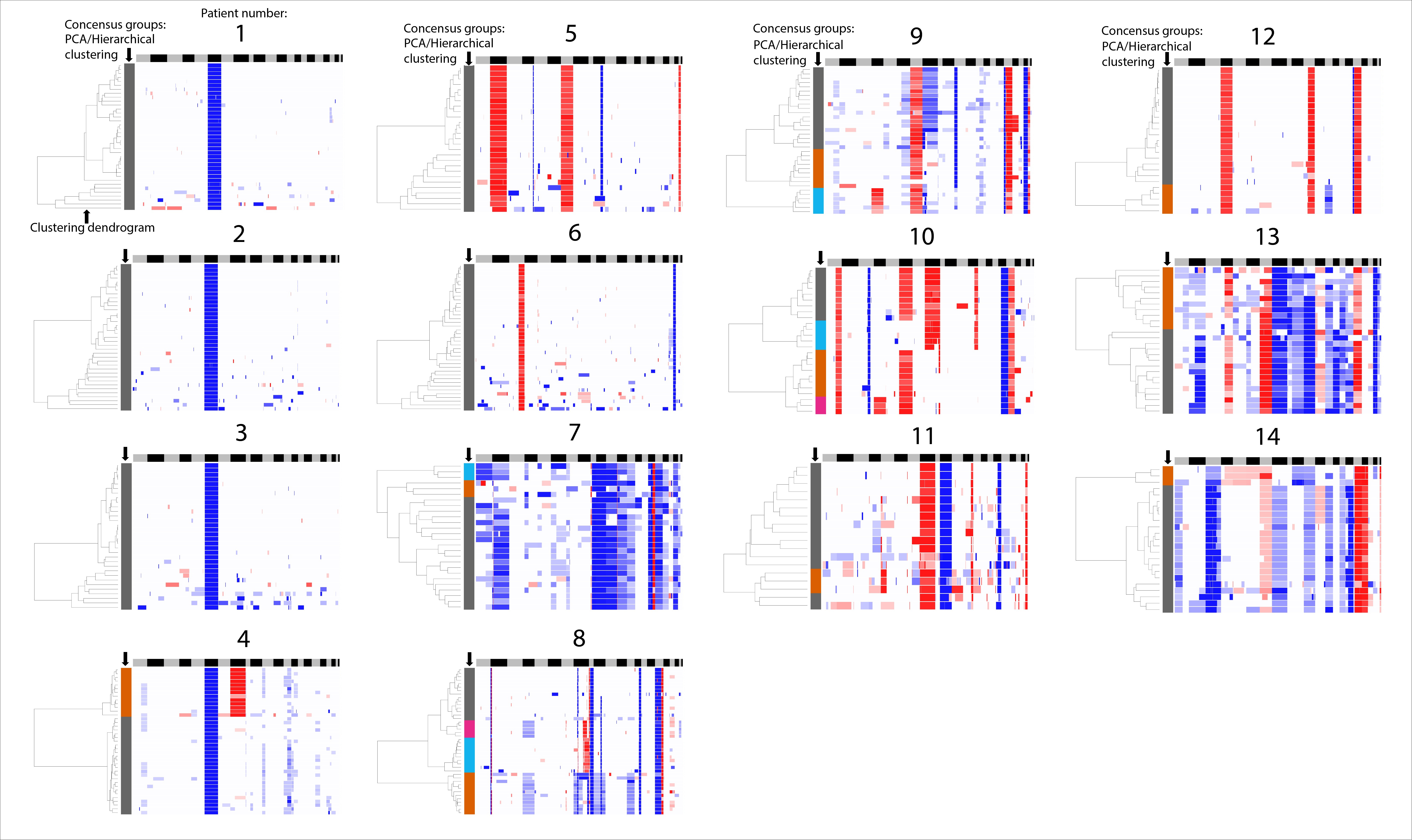
**Supplementary Fig. 3.**

**Supplementary Fig. 3: Hierarchical clustering heatmaps with dendrograms.** Panels summarize the consensus clusters based on PCA and Euclidean distances clustering.


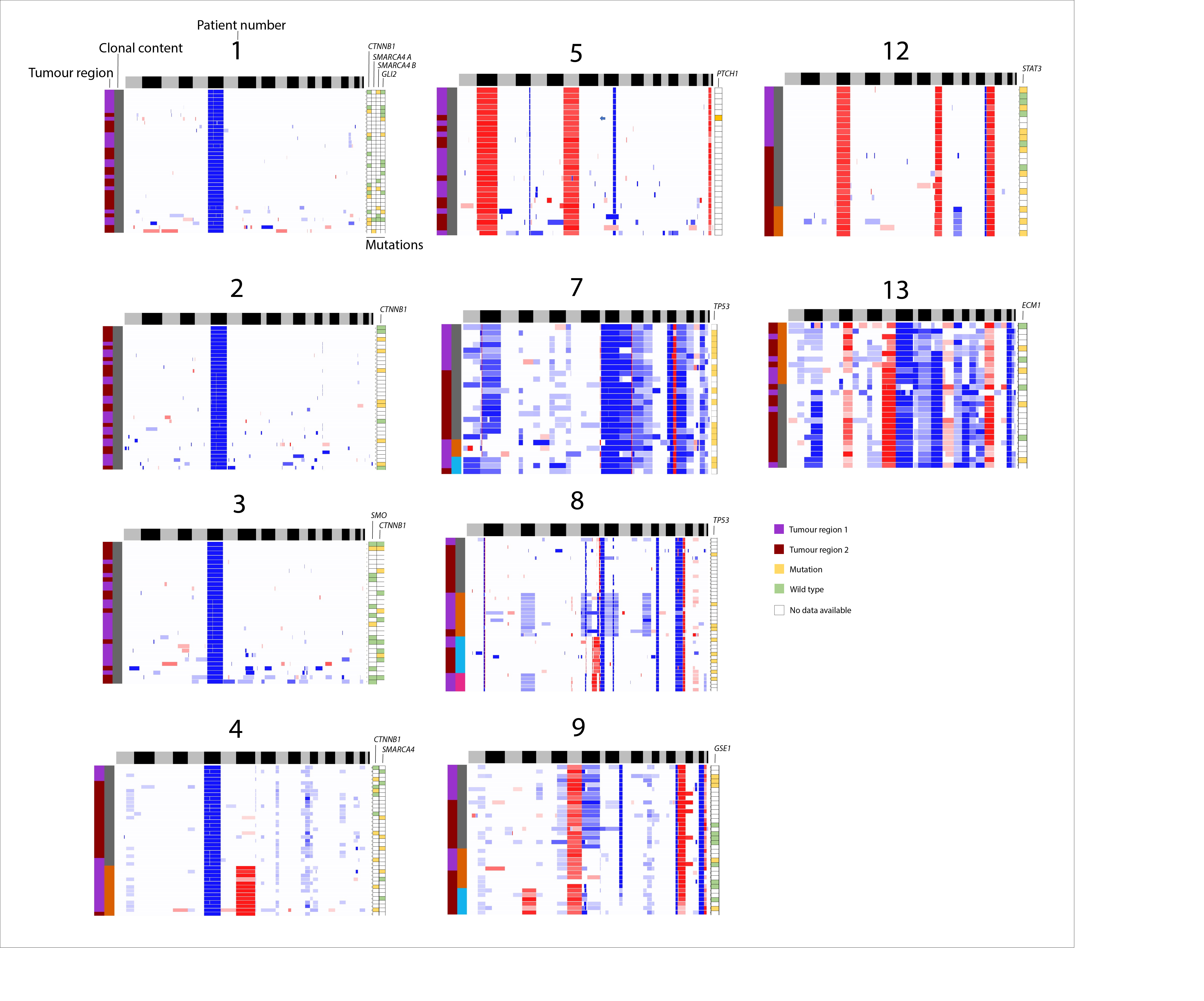
**Supplementary Fig. 4.**

**Supplementary Fig. 4: Mutation validation in the dissected clones and two tumour regions samples.** Figures summarize the information on mutations validated by Sanger sequencing in two regions of the tumour. Pink-purple and grey/orange/blue/pink columns on the left summarize the spatial origin (tumour region 1 or 2) and clonal content of individual cells, respectively. Each column on the right corresponds to a mutation, each square summarizes the status of a following mutation in a single cell. See key for colour affiliations.
